# Cancer-associated fibroblasts release exosomal microRNAs that dictate an aggressive phenotype in breast cancer

**DOI:** 10.18632/oncotarget.14752

**Published:** 2017-01-19

**Authors:** Elvira Donnarumma, Danilo Fiore, Martina Nappa, Giuseppina Roscigno, Assunta Adamo, Margherita Iaboni, Valentina Russo, Alessandra Affinito, Ilaria Puoti, Cristina Quintavalle, Anna Rienzo, Salvatore Piscuoglio, Renato Thomas, Gerolama Condorelli

**Affiliations:** ^1^ IRCCS-SDN, Naples, Italy; ^2^ Department of Molecular Medicine and Medical Biotechnology, “Federico II” University of Naples, Naples, Italy; ^3^ IEOS, CNR, Naples, Italy; ^4^ University Hospital Basel, Basel, Switzerland; ^5^ Department of Surgical and Oncology, Clinica Mediterranea, Naples, Italy

**Keywords:** exosomes, breast cancer, microenvironment, cancer-associated fibroblasts, microRNAs

## Abstract

Cancer-associated fibroblasts (CAFs) are the major components of the tumor microenvironment. They may drive tumor progression, although the mechanisms involved are still poorly understood. Exosomes have emerged as important mediators of intercellular communication in cancer. They mediate horizontal transfer of microRNAs (miRs), mRNAs and proteins, thus affecting breast cancer progression. Differential expression profile analysis identified three miRs (miRs -21, -378e, and -143) increased in exosomes from CAFs as compared from normal fibroblasts. Immunofluorescence indicated that exosomes may be transferred from CAFs to breast cancer cells, releasing their cargo miRs. Breast cancer cells (BT549, MDA-MB-231, and T47D lines) exposed to CAF exosomes or transfected with those miRs exhibited a significant increased capacity to form mammospheres, increased stem cell and epithelial-mesenchymal transition (EMT) markers, and anchorage-independent cell growth. These effects were reverted by transfection with anti-miRs. Similarly to CAF exosomes, normal fibroblast exosomes transfected with miRs -21, -378e, and -143 promoted the stemness and EMT phenotype of breast cancer cells. Thus, we provided evidence for the first time of the role of CAF exosomes and their miRs in the induction of the stemness and EMT phenotype in different breast cancer cell lines. Indeed, CAFs strongly promote the development of an aggressive breast cancer cell phenotype.

## INTRODUCTION

Breast cancer is the most common cancer in women, and is only second to lung cancer for cancer-related mortality [1]. Tumor epithelial cells coexist in carcinomas with different stromal cell types that together create the microenvironment of cancer cells. Cancer-associated fibroblasts (CAFs), the major components of tumor stroma, are active fibroblasts that, similarly to myofibroblasts, are highly heterogeneous, acquire contractile features, and express α-smooth-muscle actin (α-SMA) [2]. Active fibroblasts play similar roles in wound healing and in cancer, which may be considered as a wound that does not heal [3]. CAFs represent 80% of the resident fibroblasts in breast tumors. CAFs release high levels of growth factors, cytokines, chemokines, and metalloproteases that may affect either other stroma cells or cancer cells. Accumulated evidence indicates that they play an important role in cancer initiation, angiogenesis, invasion, and metastasis of breast cancer [4–6]. Thus, CAFs represent an attractive target for cancer therapy.

Exosomes are small (40–100 nm) vesicles that have emerged as important mediators of intercellular communication in cancer. They have been identified in most body fluids, including urine, amniotic fluid, serum, saliva, breast milk, cerebrospinal fluid, and nasal secretions [7]. Exosomes mediate local and systemic cell communication through the horizontal transfer of information, such as microRNAs, mRNAs, and proteins. Over the last decade, a number of studies has revealed that exosomes influence major tumor-related pathways, such as invasion, migration, epithelial-to- mesenchymal transition (EMT), metastasis, and therapy resistance [8–12].

MicroRNAs (miRs) are a class of non-coding 17–24 nucleotide-long RNAs that mediate post-transcriptional gene silencing. miRs are involved in many biological activities such as cell proliferation, cell differentiation, cell migration, disease initiation, and progression. Their deregulation plays an essential role in the development and progression of cancer: miRs are up- or down-regulated in malignant tissues compared to the normal counterpart, and so can be either oncogenes or tumor suppressors. Recently, microRNAs have been identified in exosomes, which can be taken up by neighboring or distant cells and subsequently promote oncogenic signaling in recipient cells upon delivery of the cargo [13–17].

Here, we analyze whether the release of CAF exosomes and their specific miR cargo could dictate an aggressive phenotype in breast cancer. Our results demonstrate that three miRs (miRs -21, -143, and -378e) are released from CAF exosomes. When loaded into breast cancer cells, they promote important tumorigenic features: stemness, EMT, and anchorage-independent cell growth. Thus, the release of CAF exosomes may be responsible for the delivery of miRs that promote oncogenic signaling in breast cancer cells.

## RESULTS

### Identification of oncogenic miRs in CAF exosomes

Breast fibroblasts were isolated from human breast biopsies for primary culture. The isolated cultures were characterized by immunocytochemistry for CK22 (pan-keratin) and Western blot analysis for e-cadherin and α-SMA (Supplementary Figure 1a, b). Exosomes were isolated from breast fibroblast-conditioned media with ExoQuick-TC and characterized by Western blot analysis for the exosomal markers CD63, CD81, Hsp70, and Alix (Supplementary Figure 1c). To identify oncogenic miRs in CAF exosomes, we conducted genome-wide expression profiling of miRs (nCounter miRNA assay, nanoString Technologies, OSU), comparing exosomal miRs derived from two breast CAF cultures (patients #3 and #4) and two normal fibroblast (NF) cultures (patients #1 and #2). We found that three miRs were significantly up-regulated in CAF exosomes respect to NF exosomes: miR-21-5p, miR-378e, and miR-143-3p (Table 1). RT-PCR was conducted to confirm the array data. Interestingly, we found that miR-143-3p was up-regulated in CAF cells as compared to NFs, but we did not observe the same for miR-21-5p or miR-378e (Supplementary Figure 2a, b, c). Furthermore, we analyzed expression levels of miRs -21, -143 and -378e in CAFs from twelve different breast cancer molecular subtypes (five luminal A, six luminal B, one HER-2), but we did not observe a significant correlation between miRs’ levels and molecular subtypes (Supplementary Figure 2d).

**Table 1:** miRNA expression profiles in NF exosomes vs CAF exosomes

	*Ratio of geom means: NF exosomes vs CAF exosomes*	*Unique id*
**1**	**0,537960845**	**hsa-miR-21-5p**
**2**	**0,703937608**	**hsa-miR-378e**
**3**	**0,768350227**	**hsa-miR-143-3p**
4	0,795360008	hsa-miR-1246
5	0,813311489	hsa-miR-1253
6	0,880781514	hsa-miR-155-5p
7	0,938786077	hsa-miR-549
8	1,032793771	hsa-miR-125b-5p
9	1,228928787	hsa-miR-1283
10	1,256584027	hsa-miR-25-3p
11	1,294286206	hsa-miR-302d-3p

Exosomes were isolated from four primary breast fibroblast cultures: two normal and two cancer-associated cultures. miRNA screening was performed by microarray, as described in Materials and Methods. Fold-change values were generated from the median expression of the miRNAs in the compared groups (NF exosomes vs CAF exosomes). Three miRs were found to be up-regulated in CAF exosomes as compared to NF exosomes.

### miRs -21, -143, and -378 expression in breast invasive carcinoma patients

**Figure 1:**
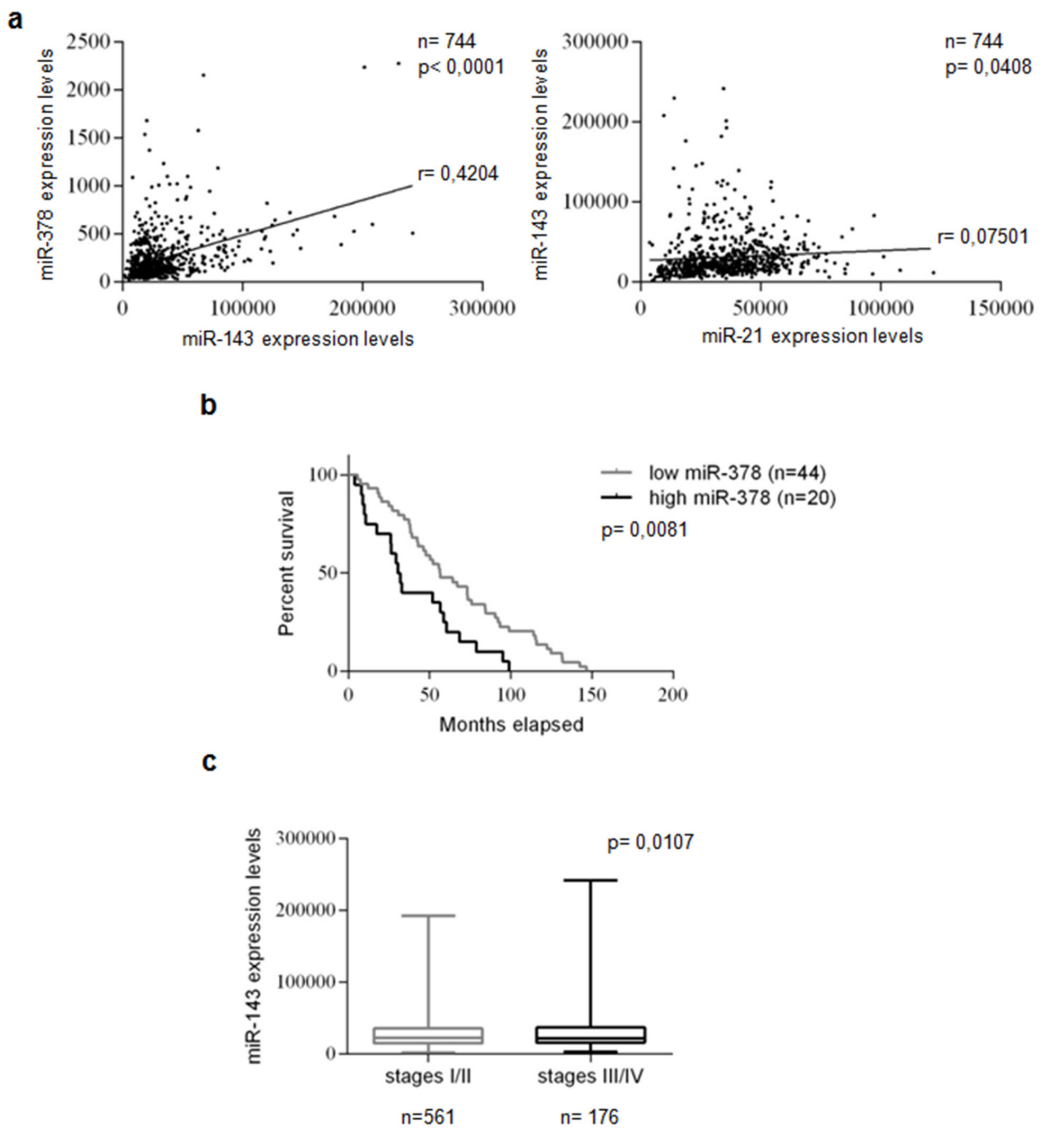
miRs -21, -143, and -378 are co-expressed, miR-378 correlates with prognosis, and miR-143 correlates with tumor stages in breast cancer miR expression was evaluated using 744 Breast Invasive Carcinoma patients from TCGA database. Statistically significant co-expression of miRs -143 and -378 levels, and of miRs -143 and -21 levels were identified in the analyzed cohort. Statistical significance calculated using Pearson's correlation analysis. P<0.05 was considered significant **a**. Kaplan-Meier survival curve: correlation between miR-378 and overall survival of 20 highly and 44 poorly miR-378-expressing breast cancer patients in TCGA database. Low miR-378 expression predicted a better prognosis in the cohort. The patients were assigned to the high or low miR-378-expressing group using the media as a threshold. P-value calculated using Log-Rank test. P<0.05 was considered significant **b**. A significant increase in miR-143 expression was identified in high (III/IV) compared to low (I/II) breast cancer stages. Statistical significance calculated using Student's t-test. P<0.05 was considered significant **c**.

We analyzed miRNA expression in a large cohort of TCGA database breast cancer patients (744 Breast Invasive Carcinoma samples). We found a statistically significant positive correlation between miR-143 and -378 (p<0.0001) and between miR-143 and -21 (p<0.05) expression (Figure 1a). These findings suggest that the co-expression of these miRs may represent a pro-oncogenic signature of Breast Invasive Carcinoma. Furthermore, Log-Rank test analysis indicated that patients with lower levels of miR-378 had longer overall survival, suggestive of a prognostic role of miR-378 (p<0.01) (Figure 1b). In addition, miR-143 expression was significantly increased in high (III/IV) compared to low (I/II) breast cancer stages (p<0.05; Figure 1c).

### Breast fibroblast-derived exosomes are transferred to T47D cells

**Figure 2:**
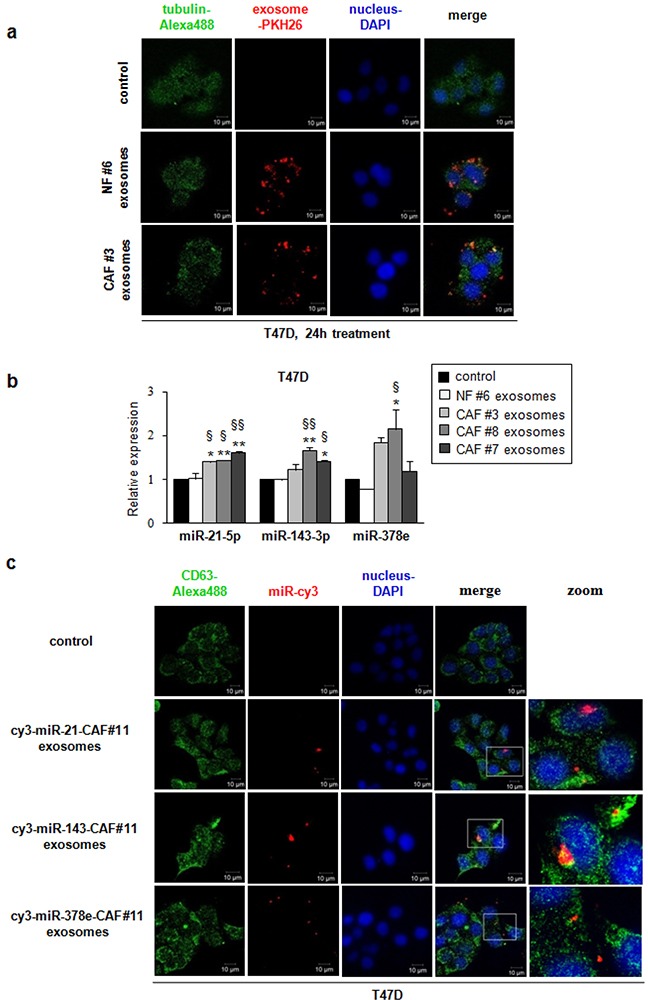
Breast fibroblast-derived exosomes are transferred to T47D cells and miRs -21, -143, and -378e are shuttled by CAF exosomes into breast cancer cells T47D cells were cultured in the absence (control) or presence of either NF- (patient #6) or CAF- (patient #3) derived PKH26-labeled exosomes for 24 hours. Exosomes were taken up by T47D cells (confocal microscopy, ×60 original magnification). T47D cells were stained using DAPI (nuclei) and ALEXA488-conjugated anti-tubulin antibody. Scale bar: 10μm **a**. T47D cells were treated 24 hours in the absence (control) or presence of either NF exosomes (patient #6) or CAF exosomes (patients #3, #7 and #8). Real Time PCR was performed to evaluate miRs -21, -143, and -378e levels. Data obtained from three independent experiments and presented as mean value ± SD. P-value calculated using one-way ANOVA followed by Bonferroni's *post hoc* testing. * p<0.05; ** p<0.01 (over control). § p<0.05; §§ p<0.01 (over NF ex) **b**. T47D cells were cultured in the absence (control) or presence of exosomes isolated from cy3-miR-CAF#11 (cy3-miRs -21, -143, -378e) for 24 hours. Cy3-labeled miRs were shuttled from CAF#11 exosomes into T47D cells (confocal microscopy, ×60 original magnification). T47D cells were stained using DAPI (nuclei) and ALEXA488-conjugated anti-CD63 antibody. Scale bar: 10μm **c**.

To examine whether fibroblast exosomes could be transferred to T47D breast cancer cells, we collected exosomes from NF- and CAF-conditioned media, and fluorescently labeled them with PKH26. T47D cells were treated with labeled exosomes for different times, and then were stained with DAPI and green ALEXA488-conjugated anti-tubulin antibody. Confocal microscopy confirmed the ability of T47D cells to take up NF- (patients #2 and #6) and CAF- (patient #3) derived exosomes (Supplementary Figure 3, Figure 2a). Notably, the uptake was greater after 24 hours (Figure 2a). To get further evidence of exosome uptake, we collected a z-stack of six images (Supplementary Figure 4). NF exosomes (patient #6, Supplementary Figure 4a) and CAF exosomes (patient #3, Supplementary Figure 4b) were clearly taken up by T47D cells after 24 hours, as demonstrated by co-localization of ALEXA488 and PKH26 signals. These results indicate that NF- and CAF-derived exosomes can be transferred to T47D cells, and may play a role in breast cancer biology.

### miRs released by CAFs are shuttled into breast cancer cells via exosomes

In order to demonstrate that CAFs vehiculate miRs -21, -143, and -378e via exosomes to breast cancer cells, we treated T47D cells with isolated CAF- or NF-exosomes (CAF patients #3, #7 and #8, NF patient #6) for 24 hours and then assessed miR levels by RT-PCR. T47D cells treated with CAF exosomes exhibited increased levels of these miRs as compared to either non-treated T47D cells or T47D cells treated with NF exosomes (Figure 2b). In addition, to visualize the transport of extracellular miRs derived from CAFs into T47D cells, we transfected CAFs (patient #11) with cy3-labeled miRs (cy3-miRs -21-5p, -143-3p, -378e) and then collected exosomes from cell media. Then, we cultured T47D cells with these exosomes. Confocal microscopy detected the signals of cy3-miRs in the cytoplasm of T47D cells (Figure 2c). Notably, as shown in z-stack images (Supplementary Figure 5a, b, c), cy3-miRs co-localized with the signals of the exosomal marker CD63. Taken together, these results suggest that CAF-secreted exosomes mediate the shuttling of miRs -21, -143, and -378e into T47D cells.

### CAF exosomes promote stemness properties, EMT phenotype, and anchorage-independent cell growth

**Figure 3:**
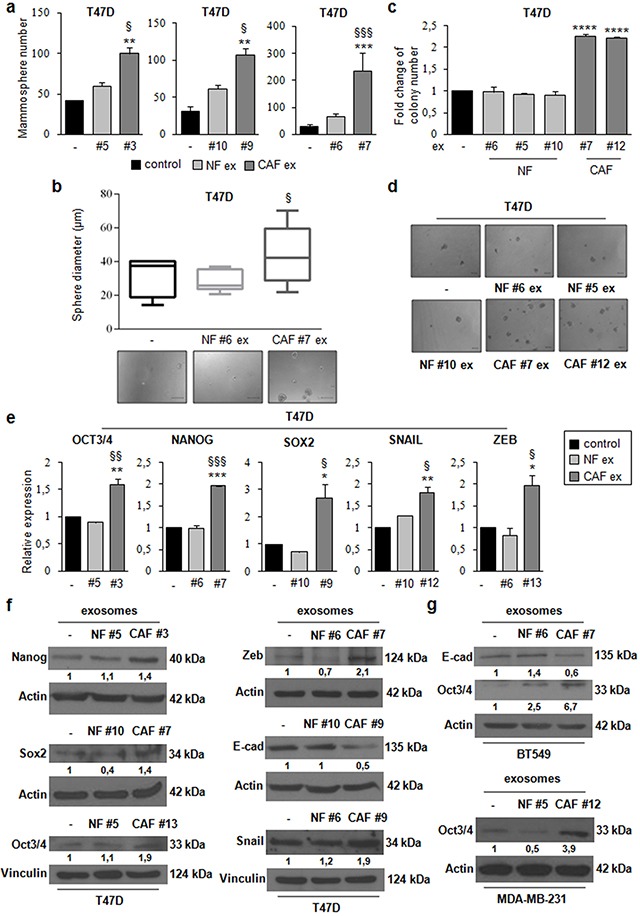
CAF exosomes promote stemness, epithelial–mesenchymal transition, and anchorage-independent cell growth T47D cells were cultured under non-adherent conditions in stem medium in the absence (control) or presence of either NF exosomes (patients #5, #6, and #10) or CAF exosomes (patients #3, #7, and #9). After four days, the capacity of cells to form spheres was assessed **a**. Sphere diameter distribution (control cells and T47D cells treated with either NF#6 ex or CAF#7 ex) for 10 representative fields. Scale bar: 100μm **b**. T47D cells were cultured in the absence (control) or presence of either NF exosomes (patients #5, #6, and #10) or CAF exosomes (patients #7, and #12). After 24h, cells were harvested and cultured in soft agar **c**. Scale bar: 100μm **d**. T47D cells were cultured in the absence (control) or presence of either NF ex (patients #5, #6, and #10) or CAF ex (patients #3, #7, #9, #12 and #13) for 24h **e**. or 48h **f**. Real Time PCR was performed to analyze oct3/4, nanog, sox2, snail and zeb mRNA levels (e). Western blot analysis was performed to evaluate nanog, sox2, oct3/4, zeb, e-cadherin and snail protein levels (f). BT549 cells were cultured in the absence (control) or presence of either NF exosomes (patient #6) or CAF exosomes (patient #7) for 48h. Western blot analysis was performed to evaluate e-cadherin and oct3/4 protein levels. MDA-MB-231 cells were cultured in the absence (control) or presence of either NF exosomes (patient #5) o CAF exosomes (patient #12) for 48h. Western blot analysis was performed to evaluate oct3/4 protein level **g**. Western blot from representative experiments is shown. Actin or vinculin were used as loading controls. The experiments were repeated at least three times (f, g). In a, b, c, e, data were obtained from three independent experiments and are presented as mean value ± SD. P-value was calculated using one-way ANOVA followed by Bonferroni's post hoc testing. * p<0.05; ** p<0.01; *** p<0,001 (over control); § p<0.05; §§ p<0.01; §§§ p<0,001 (over NF ex) (a, b, e). P-value was calculated using Student's t test. * p<0.05; ** p<0.01; *** p<0.001; **** p<0.0001 (over control) (c).

To investigate the role of CAF exosomes on stemness, we studied suspension cultures of T47D cells. Briefly, we seeded T47D cells in stem medium in non-adherent conditions in the absence or presence of NF exosomes (patients #5, #6, and #10) or CAF exosomes (patients #3, #7 and #9). After four days, we assessed the ability of cells to form mammospheres. We observed a significant increase in the number (Figure 3a) and diameter (Figure 3b) of spheres in T47D cells treated with CAF exosomes compared with either non-treated or NF exosome-treated T47D cells, indicating that CAF exosomes increase the ability to form mammospheres. We next investigated whether CAF exosomes had an impact on anchorage-independent cell growth with a soft agar assay. We found that the treatment of T47D cells with CAF exosomes (patients #7, and #12) increased the number of colonies as compared to the treatment of T47D cells with NF exosomes (#5, #6, and #10) (Figure 3c, 3d). Furthermore, the treatment of T47D cells with CAF exosomes (patients #3, #7, #9, #12 and #13), but not NF exosomes (patients #5, #6 and #10), increased the expression of stemness markers (oct3/4, nanog, sox2) and EMT markers (snail and zeb) at mRNA (Figure 3e) and protein levels (Figure 3f). In addition, the treatment of T47D cells with CAF exosomes decreased protein expression levels of the epithelial marker, e-cadherin (Figure 3f). Similar results were obtained in additional breast cancer cell lines (BT549 and MDA-MB-231) (Figure 3g). Taken together, these results clearly demonstrate that CAF exosomes foster cancer progression by promoting stemness properties, EMT phenotype, and anchorage-independent cell growth in breast cancer cells.

### miR-21, miR-143, and miR-378e promote stemness, EMT, anchorage-independent cell growth, and invasive capacity

**Figure 4:**
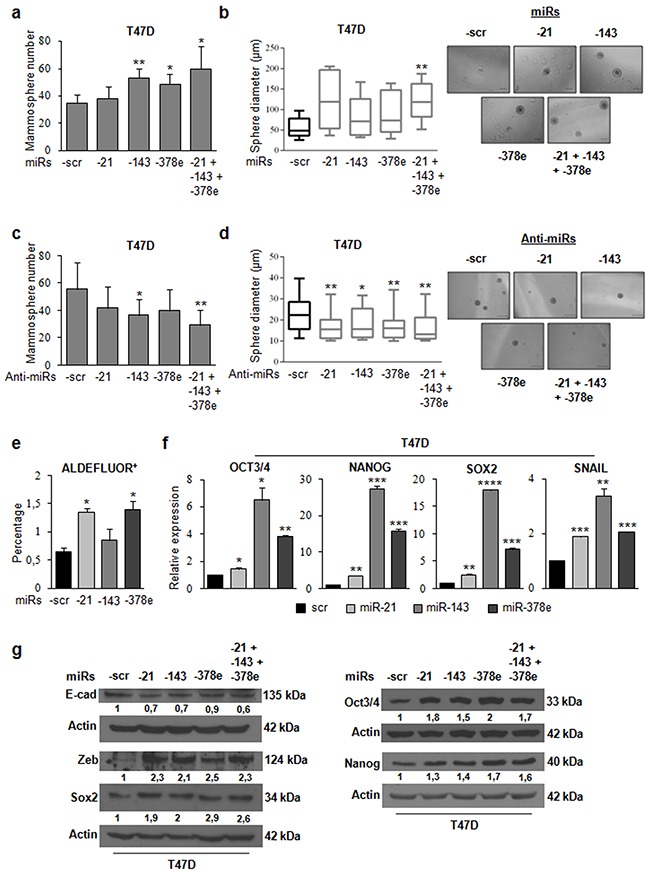
miR-21, miR-143, and miR-378e promote stemness and epithelial–mesenchymal transition T47D cells were transfected with scrambled (scr, control) or miRs -21, -143, and -378e, alone or in combination (final concentration: 100nM). After 48h, cells were harvested and cultured under non-adherent conditions in stem medium. After four days, the capacity of cells to form spheres was assessed **a**. Sphere diameter distribution for 10 representative fields. Scale bar: 100μm **b**. T47D cells were transfected with anti-miRs (alone or in combination, final concentration: 200nM) or scrambled anti-miR (anti-miR-scr, control). After 24h, cells were harvested and cultured under non-adherent conditions in stem medium. After four days, the capacity of cells to form spheres was assessed **c**. Sphere diameter distribution for 10 representative fields. Scale bar: 50μm **d**. ALDEFLUOR assays were performed in T47D cells transfected with scrambled or miRs -21, -143, and -378e for 48h **e**. T47D cells were transfected with scrambled, miRs -21, -143, or -378e for 48h **f**. or 72h **g**. Real Time PCR was performed to analyze oct3/4, nanog, sox2, and snail mRNA levels (f). In addition, T47D cells were transfected with the miRs alone or in combination for 72h. Western blot analysis was performed to evaluate nanog, sox2, oct3/4, zeb, and e-cadherin protein levels (g). In a, b, c, d, e, f, data were obtained from three independent experiments and are presented as mean value ± SD. P-value was calculated using Student's t test. * p<0.05; ** p<0.01; *** p<0.001; **** p<0.0001 (over control). Western blot analysis from representative experiments. Actin was used as loading control. The experiments were repeated at least three times (g).

**Figure 5:**
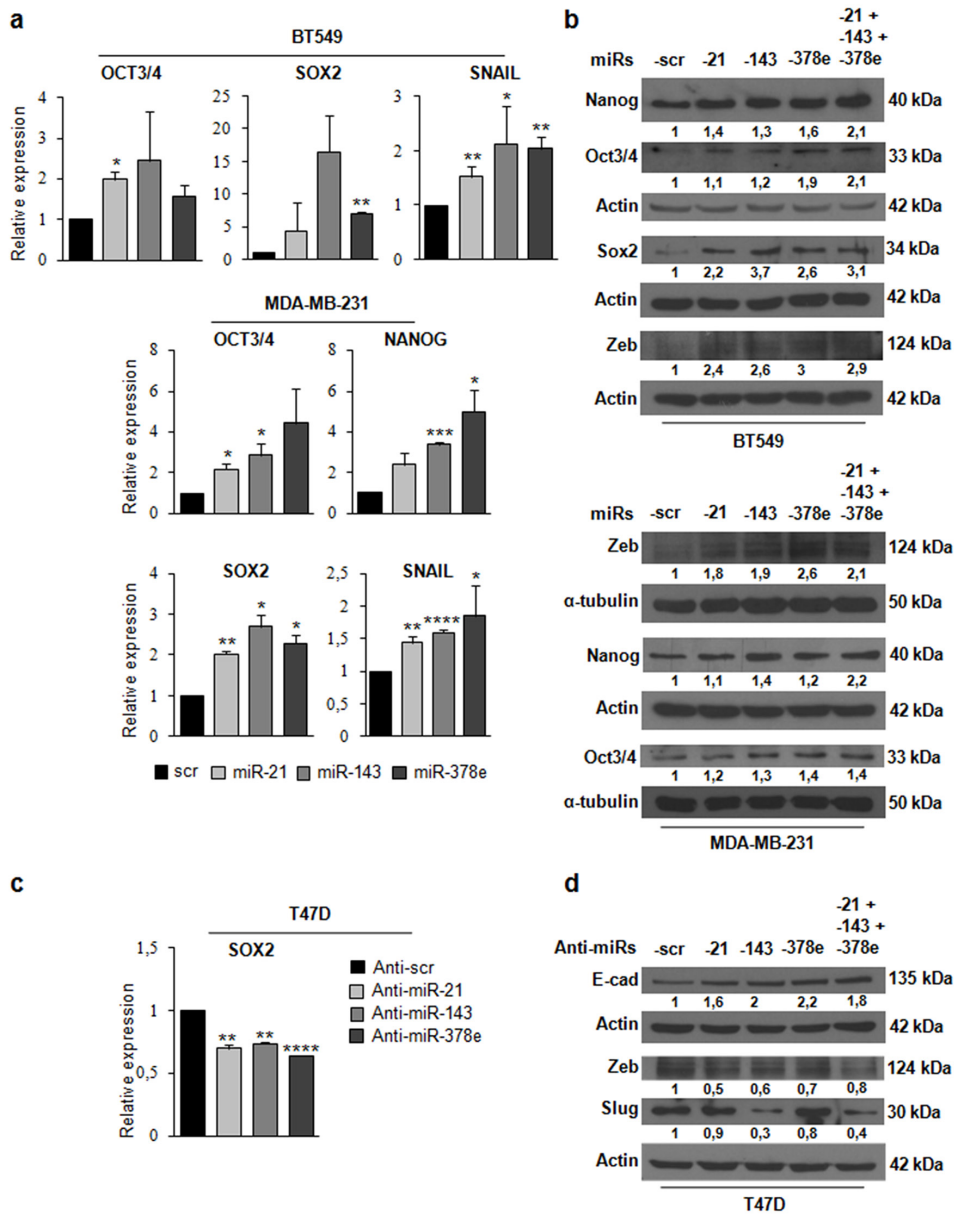
Effects of miRs-21, -143, and -378e on stemness and EMT markers in triple-negative breast cancer cells BT549 cells were transfected with miRs: scr, -21, -143, and -378e, for 24h. Real Time PCR was performed to analyze oct3/4, sox2, and snail mRNA levels **a**. In addition, BT549 cells were transfected with these miRs alone or in combination for 48h. Western blot analysis was performed to evaluate nanog, oct3/4, sox2, and zeb protein levels **b**. MDA-MB-231 cells were transfected with miRs: scr, -21, -143, and -378e, for 24h. Real Time PCR was performed to analyze oct3/4, nanog, sox2, and snail mRNA levels (a). In addition, MDA-MB-231 cells were transfected with these miRs alone or in combination for 48h. Western blot analysis was performed to evaluate zeb, nanog, and oct3/4 protein levels (b). T47D cells were transfected with anti-miRs: -scr (control), -21, -143, and -378e, for 48h. Real Time PCR was performed to analyze sox2 mRNA levels **c**. In addition, T47D cells were transfected with anti-miRs (alone or in combination; final concentration: 200nM) for 72h. Western blot analysis was performed to evaluate e-cadherin, zeb, and slug protein levels **d**. Western blot analysis from representative experiments. Actin or α-tubulin were used as loading controls. The experiments were repeated at least three times (b, d). In a and c, data were obtained from three independent experiments and are presented as mean value ± SD. P-value calculated using Student's t test. * p<0.05; ** p<0.01; *** p<0.001; **** p<0.0001 (over control).

**Figure 6:**
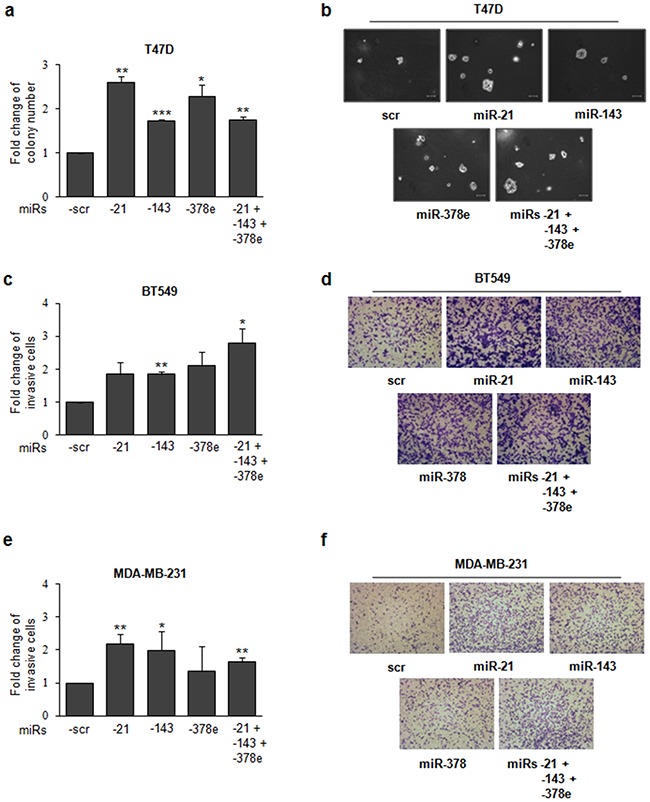
miRs -21, -143, and -378e promote anchorage-independent cell growth and invasive capacity T47D cells were transfected with scrambled miRs (scr, control), or miRs -21, -143, and -378e, alone or in combination (final concentration: 100nM). After 24h, cells were harvested and cultured in soft agar. After 3 weeks, the colonies were counted **a**. Scale bar: 100μm **b**. Invasion assays were performed in BT549 **c, d**. and MDA-MB-231 **e, f**. cells transfected with scrambled or miRs -21, -143, and -378e, alone or in combination (final concentration: 100nM). After 48h, cells were harvested and cultured in transwell plates coated with matrigel. Invasive cells were fixed with Crystal Violet solution. Percentage of invasive cells was evaluated by eluting fixed cells with 1% SDS and reading the absorbance at λ570nm. Data were obtained from three independent experiments and presented as mean value ± SD. P-value calculated using Student's t test. * p<0.05; ** p<0.01; *** p<0.001 (over control).

To investigate the biological role of the miRs included in CAF exosomes, we transfected T47D cells with miRs alone or in combination to evaluate synergic effects, and then verified whether these miRs affected the stemness phenotype of breast cancer cells through a mammosphere formation assay. MiR levels upon transfection were assessed with Real time PCR (Supplementary Figure 6a). There was a significant increase in the number (Figure 4a) and diameter (Figure 4b) of mammospheres in T47D cell cultures transfected with miRs -143, -378e, or -21/−143/−378e as compared to scrambled controls. On the contrary, T47D cells transfected with specific anti-miRs displayed a significant decrease in the number (Figure 4c) and diameter (Figure 4d) of mammospheres as compared to T47D cells transfected with anti-scrambled controls. The effects of anti-miR transfection on miR levels were verified by Real time PCR (Supplementary Figure 6b). Furthermore, we analyzed Aldehyde Dehydrogenase (ALDH) activity in T47D cells transfected with miRs -21, -143, and -378e for 48h. Interestingly, we observed an increased ALDH activity in T47D cells transfected with miRs as compared to T47D cells transfected with scrambled controls (Figure 4e). Additionally, we assessed the impact of the miRs on the expression of stem cell and EMT markers by RT-PCR and Western blot analysis. T47D cells transfected with the miRs showed increased mRNA and protein levels of stemness and EMT markers, and decreased levels of e-cadherin protein, as compared to scrambled controls (Figure 4f, 4g). Similar results were obtained in additional breast cancer cell lines (BT549 and MDA-MB-231) (Figure 5a, 5b). MiR levels in BT549 and MDA-MB-231 cells upon transfection were assessed by Real time PCR (Supplementary Figure 6a). On the contrary, we found that T47D cells transfected with specific anti-miRs exhibited decreased levels of sox2 mRNA (Figure 5c) and of EMT markers (Figure 5d). We then analyzed the effect of the miRs on anchorage-independent cell growth with a soft agar assay. We observed an increased number of colonies in T47D cells transfected with miRs as compared to scrambled controls (Figure 6a, 6b). Finally, we performed invasion assay in BT549 and MDA-MB-231 cells transfected with miR-21, miR-143 and miR-378e. Breast cancer cells transfected with miRs exhibited higher invasive capacity than cells transfected with scrambled controls (Figure 6c, 6d, 6e, 6f). Taken together, these results clearly support the idea that miRs -21, -143, and -378e, similarly to CAF exosomes, promote anchorage-independent cell growth, stemness, and EMT phenotype in breast cancer cells.

### Over-expression of CAF exosomal miRs in NF exosomes promotes stemness and EMT phenotype

**Figure 7:**
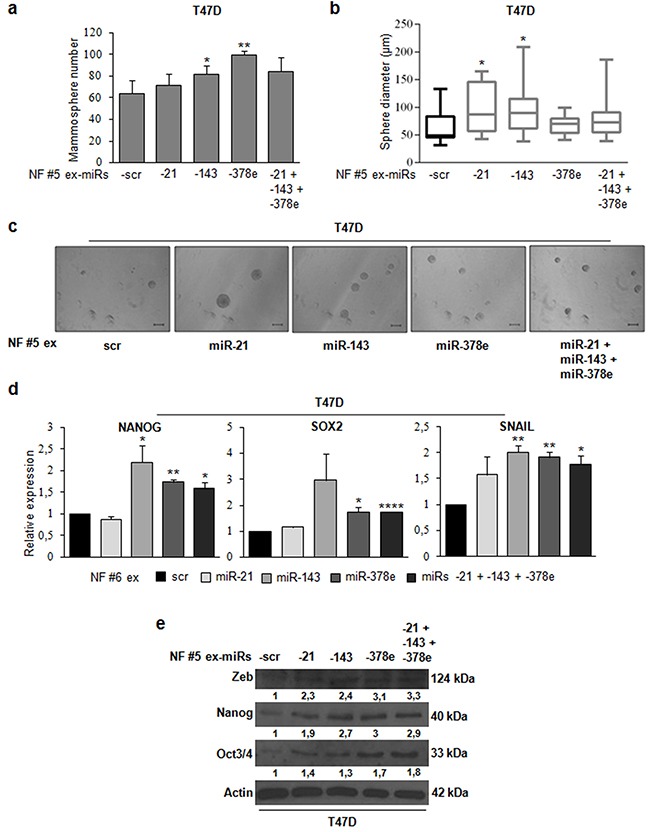
NF exosomes transfected with miRs -21, -143, -378e, similarly to CAF exosomes, promote stemness and epithelial-mesenchymal transition phenotype NF (patients #5, and #6) were cultured for 48h. Then, exosomes were isolated from NF-conditioned media with ExoQuick-TC™ solution, and transfected using Exo-Fect™ solution with scrambled miRs (scr, control), or miRs -21, -143, -378e, alone or in combination (final concentration: 130nM). T47D cells were cultured in the presence of NF exosomes (patient #5) transfected with either scrambled miRs (control) or miRs for 48h. Cells were harvested and grown under non-adherent conditions in stem medium. After four days, the capacity of cells to form spheres was assessed **a**. Sphere diameter distribution for 10 representative fields **b**. Scale bar: 100μm **c**. In addition, T47D cells were cultured in the presence of NF exosomes (patients #5, and #6) transfected with either scrambled miRs (control) or miRs for 24h **d** or 48h **e**. Real Time PCR was performed to analyze nanog, sox2, and snail mRNA (d). Western blot analysis was performed to evaluate zeb, nanog, and oct3/4 protein levels. Western blot analysis from representative experiments. Actin was used as loading control. The experiments were repeated at least three times (e). In a, b, d, data were obtained from three independent experiments and are presented as mean value ± SD. P-value calculated using Student's t test. * p<0.05; ** p<0.01; *** p<0.001; **** p<0.0001 (over control).

To demonstrate that CAF exosomes promote stemness through the identified miRs, we transfected NF exosomes with miR-21, -143, -378e, or a miR-21/−143/−378e combination with Exo-Fect. miR content in transfected exosomes was assessed by Real time PCR (Supplementary Figure 6c). When we performed a mammosphere formation assay, we observed a significant increase in the number (Figure 7a) and diameter (Figure 7b, c) of T47D spheres in cells treated with NF exosomes transfected with miRs compared to cells treated with NF exosomes containing scrambled controls. Furthermore, treated T47D cells had increased levels of stemness (oct3/4, nanog, and sox2) and EMT (snail and zeb) markers as compared to control cells (Figure 7d, e). These data clearly show that NF exosomes enriched in miRs -21, -143, and -378e acquire the ability to promote stemness properties and EMT phenotype in breast cancer cells.

## DISCUSSION

The crosstalk between cancer cells and their surrounding microenvironment is essential for tumor progression and the metastatic process. Accumulated evidence indicates that CAFs, the major components of tumor stroma, promote tumor processes such as angiogenesis, motility, metastasis, and therapy resistance in several solid tumors [4, 6, 18–21]. Tumor stroma and tumor cells communicate not only through classical paracrine signaling mechanisms (cytokines, chemokines, growth factors), but also through exosomes. Exosomes are nano-vesicles that vehiculate lipids, proteins, mRNAs, and microRNAs among cells, thereby modulating cell biology [22]. It has been recently shown that they play an essential role in cancer development, prognosis, progression, and therapy resistance [23–25]. In the context of cancer, this process entails the transfer of cancer-promoting cellular contents between cancer cells and stromal cells within the tumor microenvironment or into the circulation to act at distant sites, thereby enabling cancer progression [26]. It has been shown that melanoma-derived exosomes from the primary tumor “educated” their environment to form a pro-tumorigenic niche and programmed bone marrow-derived progenitors at the pre-metastatic site to assume a proangiogenic phenotype, thereby enhancing metastatic dissemination [11]. Furthermore, fibroblast-secreted exosomes promoted breast cancer cell migration through WNT-PCP signaling [10]. More recently, it has been demonstrated that human CAFs secreted ADAM10-rich exosomes that promoted cell motility and activated RhoA and Notch signaling in several cancer cells [27].

In order to develop therapeutic approaches, we analyzed the miR content of exosomes from breast CAFs. We describe here for the first time that CAFs promote breast cancer progression through exosome-mediated delivery of oncogenic miRs to breast cancer cells. microRNA sequencing identified increased miRs -21, -378e, and -143 levels in CAF exosomes. Immunofluorescence revealed transfer of PKH26-labeled exosomes and their miR content from fibroblasts to breast cancer cells.

miR-21 is a well-characterized oncogenic miR. In particular, its presence in breast cancer correlates with poor prognosis, regulates EMT phenotype, and promotes cancer cell proliferation and invasion [28–30]. Recently, exosomal miR-21 has been reported as an EMT promoter in oral carcinoma [25]. The tumorigenic role of miR-143 depends on the cancer type [31, 32], and recent findings suggest that it has a critical role in the tumor microenvironment of lung cancer, promoting tumor growth and neoangiogenesis [33]. MiR-378e has not been studied for an oncogenic/oncosuppressive role.

We analyzed miRNA expression in a large cohort of breast cancer patients present in the TCGA database. We found a statistically significant positive correlation between miRs-143 and -378 and between miRs-143 and -21 (p<0.05), strongly suggesting that co-expression of these miRs may represent a pro-oncogenic signature. Furthermore, Log-Rank test analysis indicated that patients with lower levels of miR-378 had longer overall survival, suggestive of a prognostic role of miR-378 (p<0.01). Finally, we found that miR-143 expression was significantly increased in high (III/IV) compared to low (I/II) breast cancer stages (p<0.05).

Many tumors, including breast cancer, are maintained by a subpopulation of cells that display stem cell properties, known as cancer stem cells (CSCs) [34, 35]. Analogous to the regulation of normal stem cells by their “niche”, CSCs are regulated by, and in turn regulate, cells within the tumor microenvironment. Several studies have provided evidence that these CSCs mediate tumor metastasis and contribute to treatment resistance and relapse [36, 37]. Recent findings have shown that the hierarchically organized cell populations are more plastic than previously imagined [38–40]. Thus, epithelial cells may dedifferentiate and thereby enter back into the stem cell pool. Thus, therapies aimed at targeting the CSCs within a tumor will not be curative if the pool of CSCs can be continuously regenerated from plastic, non-cancer stem cells capable of dedifferentiating and reentering the CSC state. Notably, accumulating evidence indicates that the expression of oct3/4, nanog, and sox2 transcription factors is strongly correlated with CSCs: knockdown of these genes decreased sphere formation and inhibited tumor development in xenograft tumor models [41, 42]. The transdifferentiation of epithelial cells into mesenchymal cells, a process known as epithelial-mesenchymal transition (EMT), is integral in development, wound healing, and stem cell behavior, and contributes pathologically to fibrosis and cancer progression. This switch is mediated by key transcription factors, including snail and zeb, and is characterized by the loss of epithelial marker expressions, such as e-cadherin. In breast carcinomas, induction of EMT promotes the generation of CSCs that are able to form mammospheres, and, similarly, CSCs isolated from tumors express EMT markers [43, 44]. In this scenario, discovering the underlying mechanisms that promote dedifferentiation processes could provide new targets for therapeutic strategies.

In the present study, we demonstrate that CAF exosomes and their miRs promote dedifferentiation of breast cancer cells. For the first time, we provide evidence that CAF exosomal miRs induce stemness and EMT phenotype in breast cancer cells. Moreover, normal fibroblast exosomes transfected with these miRs promoted stemness and EMT, similarly to CAF exosomes. Interestingly, we found that breast cancer cells treated with CAF exosomes or transfected with miR-143 and miR-378e showed increased capacity to form mammospheres. Importantly, normal fibroblast exosomes, once transfected with miRs -378e, and -143, similarly to CAF exosomes, promoted the mammosphere formation. On the contrary, breast cancer cells co-transfected with combinations of specific anti-miRs displayed a strong reduction of these effects, thereby underlying a good strategy to prevent dedifferentiation of breast cancer cells. Finally, we found that CAF exosomes and their miRs increased anchorage-independent cell growth in breast cancer cells. Our data show that the identified miRs does not act in a synergic way, but together they may favor an aggressive phenotype: miR-21 seems to be the best inducer of anchorage-independent cell growth and increases stemness and EMT markers, while miR-143 and miR-378e are the strongest promoters of stemness. In summary, we conclude that CAFs regulate the development of an aggressive phenotype in breast cancer cells through exosome-mediated delivery of oncogenic miRs.

## MATERIALS AND METHODS

### Primary and continuous cells and mammosphere cultures

Breast cancer cell lines, T47D, BT549, MDA-MB-231 from ATCC, were grown in RPMI-1640 medium. Primary cultures of fibroblasts, deriving from patients treated at the Clinica Mediterranea (Naples, Italy), were grown in Dulbecco's Modified Eagle's Medium/Nutrient F12-Ham (DMEM-F12). Media were supplemented with 10% heat-inactivated fetal bovine serum (FBS) and 100 U/ml penicillin/streptomycin. All media and supplements were from Sigma-Aldrich (Milan, Italy).

For mammosphere cultures, single-cell suspensions were plated at a density of 1,000 cells/ml in Corning ultra-low attachment multi-well plates. Cells were grown in stem medium: serum-free DMEM-F12 (Sigma, Milan, Italy) supplemented with B27 (Life technologies, Milan, Italy), 20ng/ml EGF (Sigma, Milan, Italy), 10ng/ml bFGF (BD Biosciences, Milan, Italy), and 1X antibiotic-antimycotics (Life technologies, Milan, Italy). After 4-7 days, mammospheres appeared as spheres of floating viable cells.

### Isolation of primary cell cultures from human breast biopsies

Human breast biopsies (samples from the Clinica Mediterranea) were cut by mechanical fragmentation with sterile scissors and tongs. Extracellular matrix was digested with collagenase (Sigma-Aldrich) overnight under continuous agitation at 37°C. The drawn cellular suspensions were separated on the basis of their weights by two different centrifugations: the first one at 500 rpm for 5 minutes to obtain epithelial cell pellets, the second one at 1200 rpm 5 minutes to obtain fibroblast pellets.

### Immunocytochemistry

The separation between epithelial cells and fibroblasts from human breast biopsies was confirmed by the cell block technique (Shandon cytoblock kit) followed by immunocytochemistry. A primary antibody was used for CK22 (pan-keratin), a known epithelial marker, in order to discriminate epithelial cells from fibroblasts.

### Exosome isolation

Exosomes were isolated from cell culture media of primary fibroblasts that were grown in DMEM-F12 (Sigma-Aldrich) supplemented with 10% Exo-FBS (FBS depleted of exosomes, SBI, System Biosciences) and 1X antibiotic-antimycotics (Life technologies, Milan, Italy). Culture media were centrifuged at 3000 g for 15 minutes at 4°C or room temperature to remove cellular debris. The supernatants were transferred to sterile tubes in an appropriate volume of the ExoQuick-TC™ Exosome Isolation Reagent (SBI, System Biosciences), considering 2 ml of ExoQuick-TC™ solution for every 10 ml of cell culture medium. Then, the tubes were subjected to mild agitation until the separation between the two phases was no longer visible. Finally, the tubes were kept at 4°C O/N (12 hours at least were necessary). The following day, the tubes were centrifuged firstly at 1500 g for 30 minutes, and then at 1500 g for 5 minutes (at 4°C or RT). At the end of the steps, white/beige exosomal pellets appeared.

### RNA extraction and real time PCR

Total RNA (microRNAs and mRNAs) was extracted using Trizol (Invitrogen, Milan, Italy) according to the manufacturer's protocol. Reverse transcription of total RNA was performed starting from equal amounts of total RNA/sample (150/500ng) using miScript reverse Transcription Kit (Qiagen, Milan, Italy) for miR analysis, and using SuperScript® III Reverse Transcriptase (Invitrogen, Milan, Italy) for mRNA analysis. Quantitative analysis of miR-21, miR-143, miR-378e and RNU6A (as an internal reference) was performed by Real Time PCR using specific primers (Qiagen, Milan, Italy) and miScript SYBR Green PCR Kit (Qiagen, Milan, Italy). Real Time PCR was also used to assess the mRNAs of nanog, oct3/4, sox2, snail, zeb, and β-actin (as an internal reference), using iQ™ SYBR Green Supermix (Bio-Rad, Milan, Italy). The primer sequences were: nanog-fw: 5′-CAAAGGCAAACAACCCACTT-3′, nanog-rv: 5′-TCTGGAACCAGGTCTTCACC-3′, oct3/4-fw: 5′-CGAAAGAGAAAGCGAACCAG-3′, oct3/4-rv: 5′-GCCGGTTACAGAACCACACT-3′, sox2-fw: 5′-GCACATGAACGGCTGGAGCAACG-3′, sox2-rv: 5′-TGCTGCGAGTAGGACATGCTGTAGG-3′, snail-fw: 5′-AGTGGTTCTTCTGCGCTACT-3′, snail-rv: 5′-GGGCTGCTGGAAGGTAAACT-3′, zeb-fw: 5′-CCAGACAGTGTTACCAGGGAGGAG-3′, zeb-rv: 5′-TGCCCTTCCTTTCCTGTGTCATCC-3′, actin-fw: 5′-TGCGTGACATTAAGGAGAAG-3′, actin-rv: 5′-GCTCGTAGCTCTTCTCCA-3′.

The reaction for detection of mRNAs was performed in this manner: 95°C for 5′, 40 cycles of 95°C for 30′’, 62°C for 30′’ and 72°C for 30′’. The reaction for detection of miRs was performed in this manner: 95°C for 15′, 40 cycles of 94°C for 15′’, 55°C for 30′’ and 70°C for 30′’. All reactions were run in triplicate. The threshold cycle (CT) is defined as the fractional cycle number at which the fluorescence passes the fixed threshold. For relative quantization, the 2(−ΔΔCT) method was used. Experiments were carried out in triplicate for each data point, and data analysis was performed by using Applied Biosystems StepOnePlus™ Real-Time PCR Systems.

### NCounter miRNA assay

Exosomes were isolated from four different samples of primary fibroblasts: two normal and two cancer-associated fibroblasts. Then, exosomal RNA was extracted in order to perform an “nCounter miRNA assay” (nanoString Technologies, OSU, Columbus, OHIO). Eight-hundred human miRs were digitally detected and counted in a single reaction without amplification. System performance consisted of 6 positive miRNA assay controls, 6 negative miRNA assay controls, and 5 mRNA housekeeping controls. 100ng of purified total RNA was used as starting material. miR expression levels were measured calculating the ratio of geom. means (NF exosomes vs CAF exosomes).

### TCGA data analysis

The collection of data from The Cancer Genome Atlas (TCGA) platform was compliant with laws and regulations for the protection of human subjects, and necessary ethical approvals were obtained. Analysis of all data was done using GraphPad Prism 6 (San Diego, CA, USA). For our analysis, we downloaded BCGSC IlluminaHiSeq_miRNASeq (level 3) along with clinical information from the TCGA database in July 2015.

### Cell and exosome transfection

For transient over-expression/downregulation of miRs, cells at 50% confluence were transfected using Oligofectamine (Invitrogen, Milan, Italy) and 100nM of pre-miR-21, pre-miR-143-3p, pre-miR-378e, or scrambled pre-miR; or 200nM of anti-miR-21, anti-miR-143-3p, anti-miR-378e, or scrambled anti-miR (Ambion®, Life Technologies). For miR over-expression, exosomes isolated with ExoQuick-TC™ solution were transfected using Exo-Fect™ Exosome Transfection Reagent (SBI, System Biosciences) and 130nM of pre-miR-21, pre-miR-143-3p, pre-miR-378e, or scrambled pre-miR.

### Transfection of CAFs with Cy3-labeled-miRs

CAFs were transfected with 10nM of Cy3-labeled miRs (miR-21-5p, miR-143-3p, miR-378e, Tebu-bio, San Diego, CA, USA) using Oligofectamine (Invitrogen, Milan, Italy). Six hours after transfection, cells were washed twice with PBS, and the culture media switched to fresh DMEM-F12 1% A/A, 10% exo-FBS. After incubation for a day, the culture media were collected and used for exosome preparation.

### Protein isolation and western blotting

Cells were washed twice in ice-cold PBS, and lysed in JS buffer (50mM HEPES ph 7.5 containing 150mM NaCl, 1% Glycerol, 1% Triton X100, 1.5mM MgCl_2_, 5mM EGTA, 1mM Na3VO4, and 1X protease inhibitor cocktail). Exosomes were lysed in RIPA lysis buffer (Sigma-Aldrich) containing protease and phosphatase inhibitors (Roche Diagnostics GmbH, Mannheim, Germany). Protein concentration was determined by the Bradford assay (Bio-Rad, Milan, Italy) using bovine serum albumin (BSA) as the standard, and equal amounts of proteins were analyzed by SDS-PAGE (10%, 12% or 15% acrylamide). Gels were electroblotted onto nitrocellulose membranes (GE Healthcare, Milan, Italy). For immunoblot experiments, membranes were blocked for 1 hour with 5% non-fat dry milk in Tris Buffered Saline (TBS) containing 0,1% Tween-20, and incubated at 4°C overnight with primary antibodies. Detection was performed by peroxidase-conjugated secondary antibodies using the enhanced chemiluminescence system (Thermo, Euroclone, Milan, Italy). Primary antibodies used were: anti-β-actin and anti-α-tubulin from Sigma-Aldrich (Milan, Italy), anti-vinculin, anti-α-SMA, anti-e-cadherin, anti-zeb, anti-oct3/4, anti-nanog, anti-sox2, anti-slug, anti-snail, anti-CD63, anti-hsp70, anti-alix, anti-CD81 from Santa Cruz Biotechnology (CA, USA). ImageJ software was used to provide WB quantification.

### Immunofluorescence analysis

1) Uptake of fibroblast exosomes by T47D cells. Once isolated, exosomes were labeled with PKH26, a red fluorescent cell membrane linker (Sigma-Aldrich). Briefly, exosomes were stained 5 minutes at RT with PKH26 (diluted 1:1000 in PBS), then 1ml of BSA 1% was added to the tubes and mixed. Finally, the tubes were centrifuged at 1500 g for 5 minutes and pellets were put in 500μl of PBS. For immunofluorescence, cells grown on glass coverslips were treated at different incubation times with PKH26-exosomes, washed six times with PBS and fixed with 4% paraformaldehyde in PBS for 20 minutes at room temperature. The coverslips were washed three times in PBS. Then, cells were permeabilized with PBS 0.5% Triton X-100 for 15 minutes at room temperature, and blocked in PBS 1% bovine serum albumin (BSA) for 15 minutes. Cells were incubated with anti-tubulin antibody (Santa Cruz), diluted in PBS 1% BSA, for 1 hour at 37°C. Coverslips were washed 3 times with PBS and treated with Alexa Fluor 488 Goat Anti-Mouse IgG (Invitrogen) for 30 minutes at 37°C. Coverslips were washed and mounted with Gold antifade reagent with DAPI (Invitrogen). The cells were visualized by confocal microscopy. 2) Shuttling assays for fluorescently-labeled miRNAs. After a day from the transfection of CAFs with Cy3-labeled-miRs, exosomes were isolated from cell media, and T47D cells grown on glass coverslips were treated for 24 hours with the isolated exosomes. For immunofluorescence, it was used the same protocol as above, but cells were incubated with anti-CD63 primary antibody (Santa Cruz), then with Alexa Fluor 488 Goat Anti-Rabbit IgG (Invitrogen).

### Mammosphere formation assay

T47D cells were suspended 1000 cells/well in 1 ml of stem medium and plated into ultra-low attachment 48-well plates in presence of normal fibroblast or CAF exosomes. Alternatively, T47D cells were transfected with miRs or anti-miRs for 48 hours or 24 hours, respectively. Then, 1000 cells/well were suspended in 1 ml of stem medium and plated into ultra-low attachment 48-well plates. After 4-7 days, the formed mammospheres were counted, and the diameters were measured.

### ALDEFLUOR assay

The ALDEFLUOR assay was carried out according to manufacturer's (Stemcell Technologies) guidelines. Briefly, dissociated single cells were suspended in Aldefluor assay buffer containing an ALDH substrate, bodipy-aminoacetaldehyde (BAAA) (5μl per milliliter of sample), and incubated for 40 min at 37°C. A fraction of cells was incubated under identical conditions in the presence of ALDH inhibitor (10μl per milliliter of sample), diethylaminobenzaldehyde (DEAB). Cells were analyzed using a BD Accuri™ C6 (BD Biosciences) flow cytometer and analyzed using the BD Accuri™ C6 software.

### Soft-agar assay

10^4^ cells were plated in 60mm dishes in a solution containing Dulbecco's modified Eagle's medium 2× (Sigma, St Louis, MO, USA), TPB Buffer (Difco, BD, Franklin Lakes, NJ, USA), and 1.25% of Noble Agar (Difco, BD, Franklin Lakes, NJ, USA). Briefly, cells were harvested and counted, then a layer of 7ml with the solution containing Noble Agar were left to polymerize on the bottom of the dishes. Finally, cells were resuspended in 2ml of same solution and plated. Cells were left grown for 3 weeks in the incubator.

### Invasion assay

In vitro invasion assay was performed in 24-well Corning Multiwells 6.5mm Transwell® with 8.0μm Pore Polycarbonate Membrane Inserts. The lower chamber was filled with 600μl of RPMI medium supplemented with 10%FBS and 1% antibiotic-antimycotics. The top of the upper wells was coated with 25μl of Matrigel Basement Membrane Matrix (BD) diluted 1:6 in RPMI free medium and allowed to dry out at 37°C at least 4h. Then, 50.000 cells, previously transfected with miRs for 48h, were plated in RPMI free medium in the upper chamber. Cells were incubated at 37°C for 20h. Then, cells on the upper surface of the membrane were removed mechanically by wiping with a cotton swab, and the cells remaining on the lower side of the membrane were fixed and stained with Crystal Violet solution. Percentage of invasive cells was evaluated by eluting Crystal Violet solution with 1% SDS and reading the absorbance at λ570nm.

### Statistical analysis

All experiments were repeated at least three times. Continuous variables are given as mean ± standard deviation. For comparisons between two groups, the Student's t test was used to determine differences between mean values for normally distributed. Comparisons among more than three groups were determined by one-way ANOVA followed by Bonferroni's post hoc testing. All data were analyzed for significance using GraphPad Prism 6 software (San Diego, CA, USA). P values less than 0.05 were considered significant.

## SUPPLEMENTARY MATERIALS FIGURES


