# Supplementary material for: Cancer-associated fibroblasts release exosomal microRNAs that dictate an aggressive phenotype in breast cancer
Source: Oncotarget. 2017 Jan 19;8(12):19592–608. doi: 10.18632/oncotarget.14752 (PMC5386708; doi:10.18632/oncotarget.14752)
Supplement: Supplementary file 1 [file oncotarget-08-19592-s001.pdf]

Cancer-associated fibroblasts release exosomal microRNAs that dictate an aggressive phenotype in breast cancer

SUPPLEMENTARY FIGURES

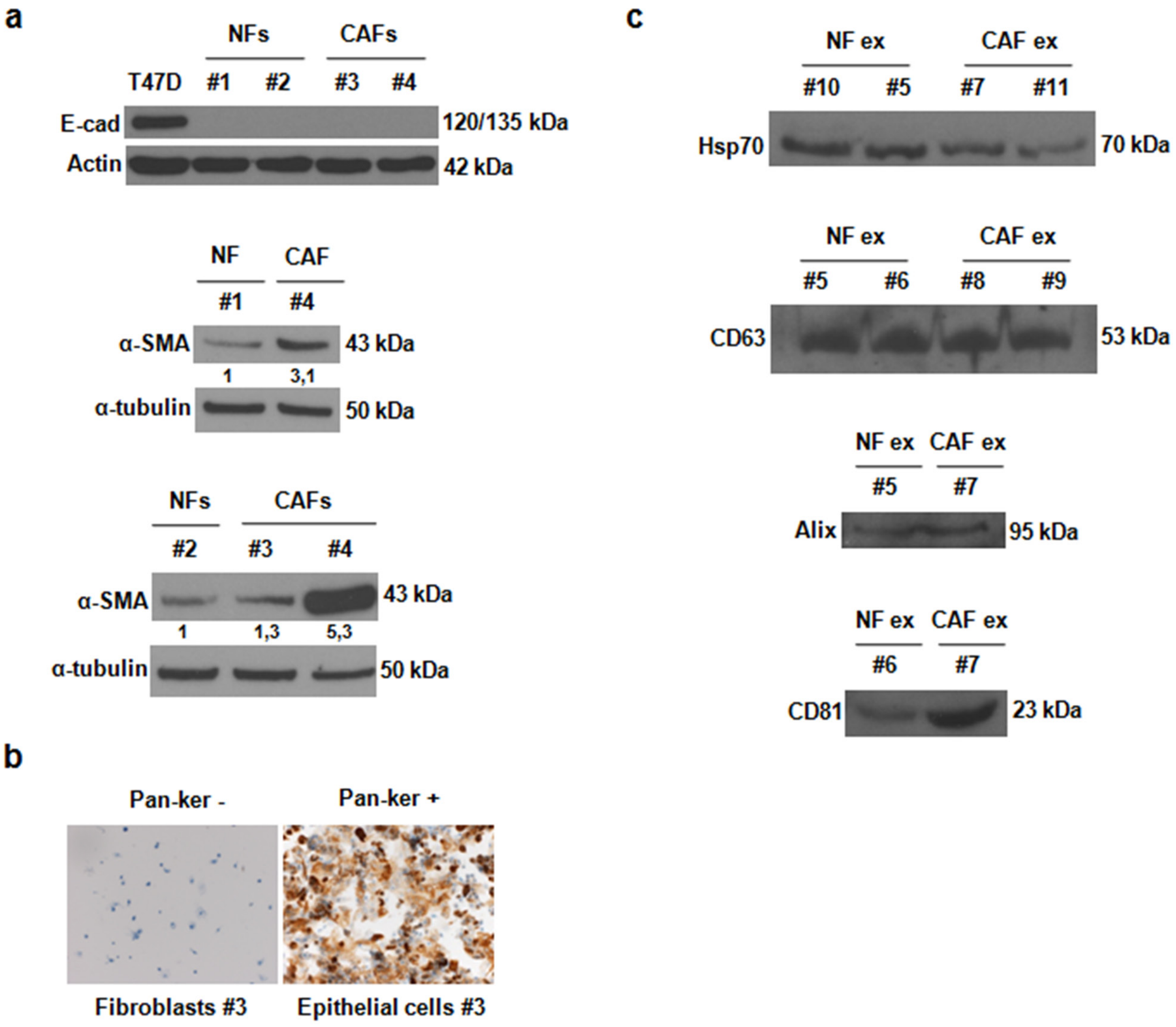

Supplementary Figure 1: Characterization of human breast fibroblasts and fibroblast exosomes. Western blot analysis was performed to evaluate e-cadherin and α-SMA protein levels. All the fibroblasts were e-cadherin negative compared to the positive control, T47D cells. Cancer-associated fibroblasts (patients #3 and #4) overexpressed α-SMA as compared to normal fibroblasts (patients #1 and #2). Actin and α-tubulin were used as loading controls. Western blot analysis is from representative experiments **a.** The correct separation of cell populations from breast biopsies was confirmed with the cell block technique (Shandon cytoblock kit) followed by immunocytochemistry for the epithelial marker CK22 (pan-keratin). In **b.** patient #3 biopsy: fibroblasts were CK22 negative, whereas epithelial cells were CK22 positive. Western blot analysis was performed to evaluate exosomal marker protein levels. Exosomal proteins (Hsp70, CD63, Alix, CD81) were expressed in NF exosomes (NF ex) and in CAF exosomes (CAF ex). Western blot analysis is from representative experiments **c.**

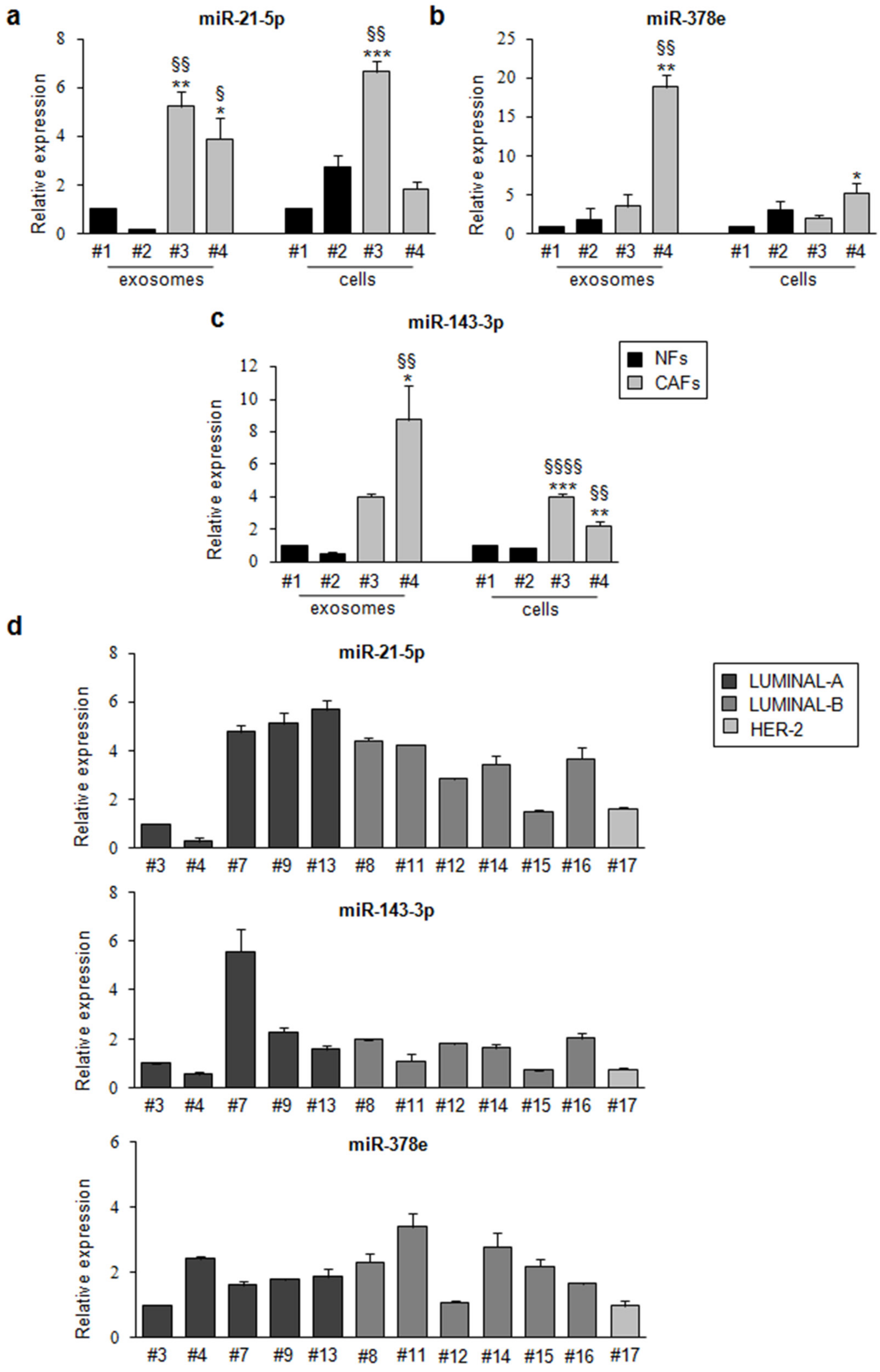

Supplementary Figure 2: Array validation. Real Time PCR of miR-21-5p, miR-378e, and miR-143-3p expression levels in 2 NF and 2 CAF exosomes and cells. miR-21, miR-378e, and miR-143 were up-regulated in CAF versus NF exosomes. miR-143 was up-regulated in CAF versus NF cells. Data obtained from three independent experiments and presented as mean value ± SD. P-value calculated using Student's t test. * $p<0.05$; ** $p<0.01$; *** $p<0.001$ (over NF #1). § $p<0.05$; §§ $p<0.01$; §§§ $p<0.001$; §§§§ $p<0.0001$ (over NF #2) **a, b, c.** Real Time PCR of miR-21-5p, miR-143-3p, and miR-378e expression levels in twelve CAFs of different breast cancer molecular subtypes (five luminal A, six luminal B, one HER-2) **d.**

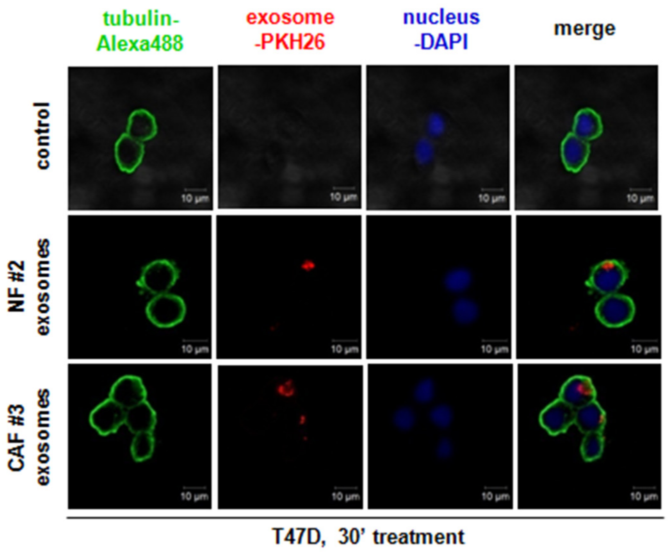

Supplementary Figure 3: Breast fibroblast-derived exosomes are transferred to T47D cells. T47D cells were cultured in the absence (control) or presence of either NF- (patient #2) or CAF- (patient #3) derived PKH26-labeled exosomes for 30 minutes. Exosomes were taken up by T47D cells (confocal microscopy, ×60 original magnification). T47D cells were stained using DAPI (nuclei) and ALEXA488-conjugated anti-tubulin antibody. Scale bar: 10µm.

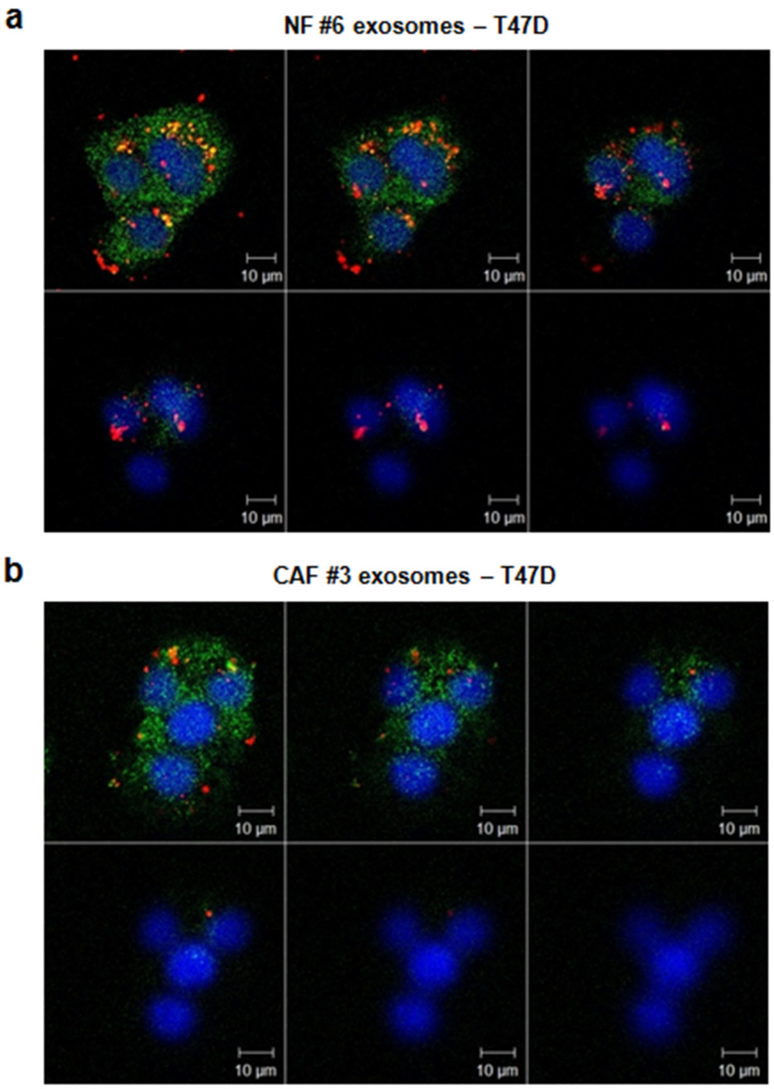

Supplementary Figure 4: Z-stack images of exosome uptake. T47D cells were cultured in the presence of fibroblast PKH26-labeled exosomes for 24 hours and stained with DAPI (nuclei) and ALEXA488-conjugated anti-tubulin antibody. Six images for each point were acquired, with slice thickness of 1µm. NF exosomes (patient #6, **a.**) and CAF exosomes (patient #3, **b.**) were taken up by T47D cells, as demonstrated by the co-localization of ALEXA488 and PKH26 signals. Confocal microscope (original magnification, ×60). Scale bar: 10µm.

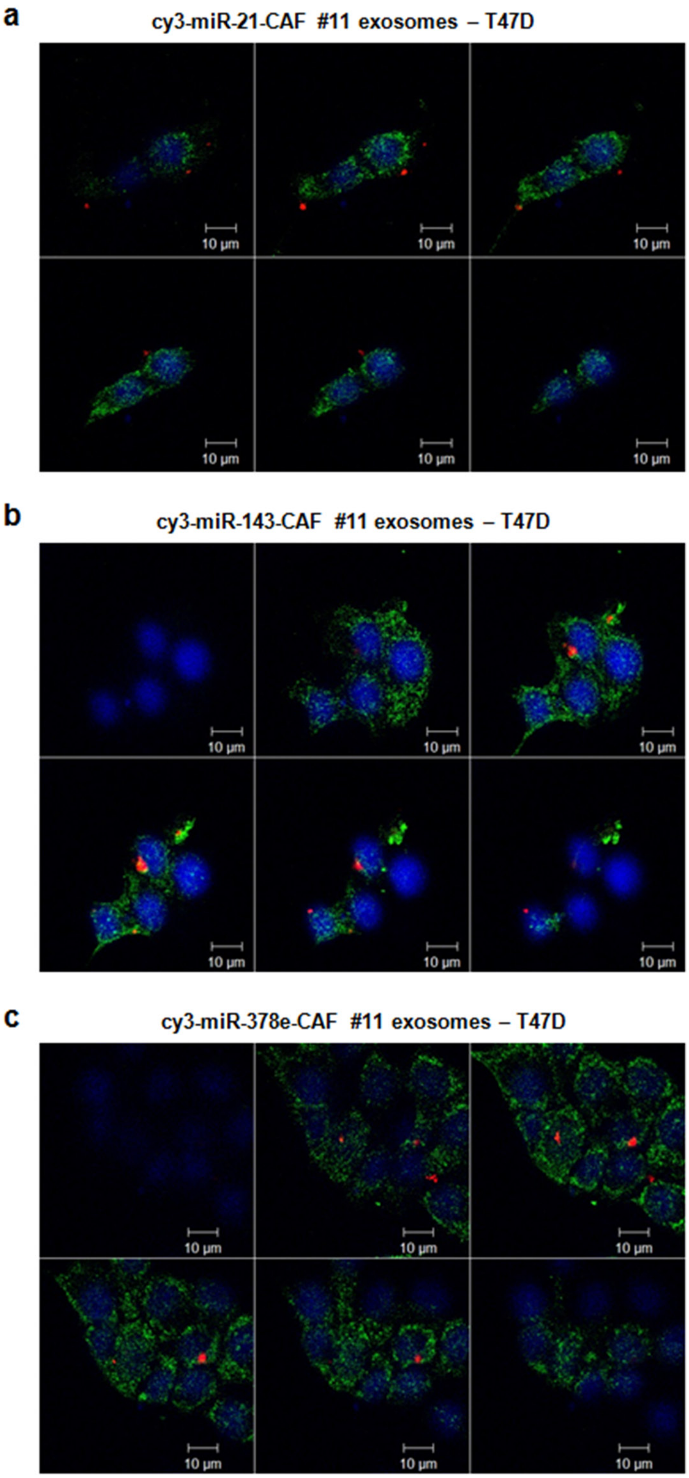

Supplementary Figure 5: Z-stack images of cy3-labeled miRs shuttled from CAF exosomes into T47D cells. T47D cells were cultured with exosomes isolated from cy3-miR-CAF#11 (cy3-miRs -21, -143, -378e). The treated cells were stained with DAPI (nuclei) and ALEXA488-conjugated anti-CD63 antibody. Six images for each point were acquired, with slice thickness of 1µm. Cy3-labeled miRs were shuttled by CAF#11 exosomes into T47D cells, as shown by the co-localization of cy3-miRs and Alexa488-CD63 signals. Confocal microscopy (original magnification, ×60). Scale bar: 10µm.

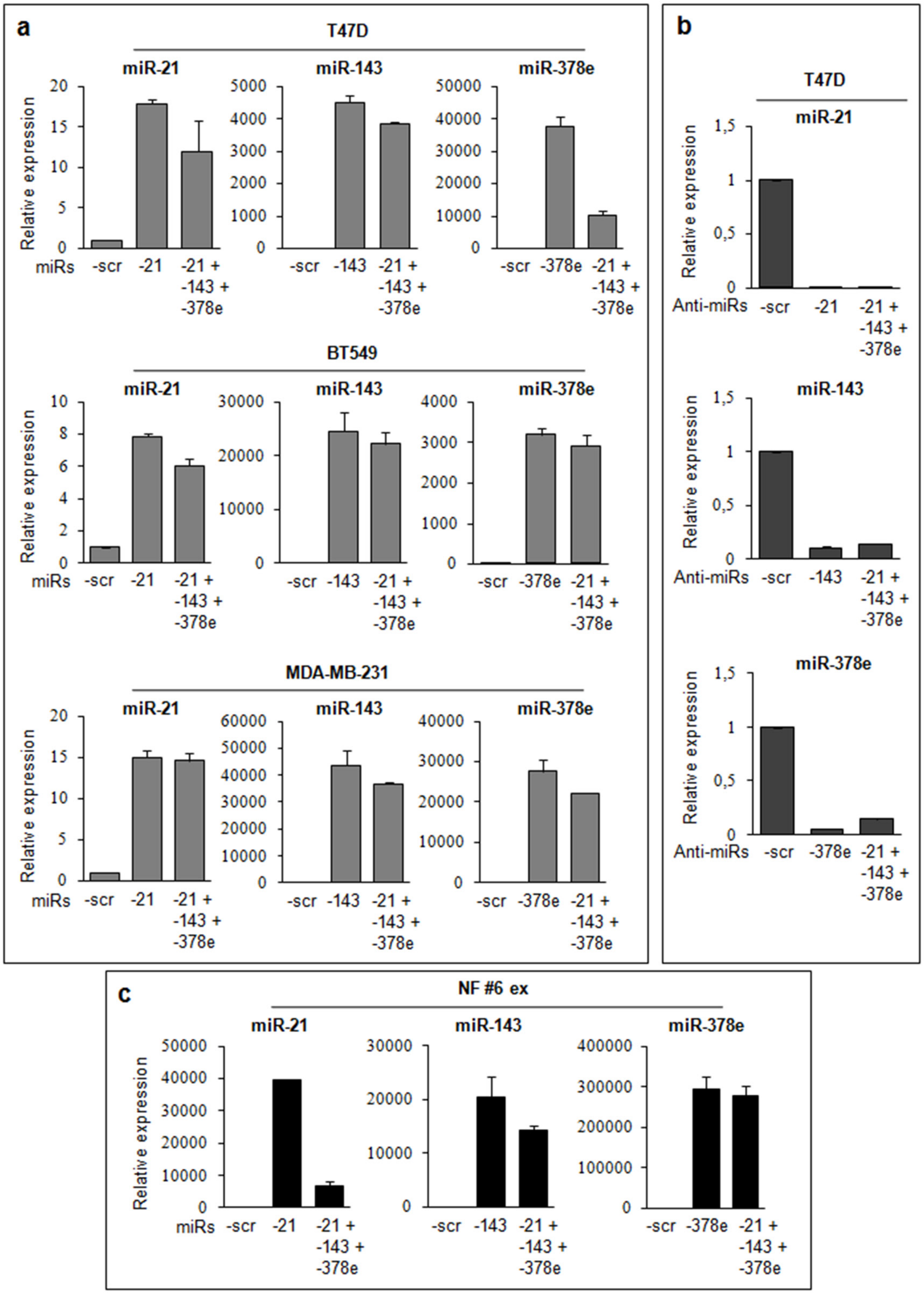

Supplementary Figure 6: Real time PCR of miR-21, miR-143, and miR-378e expression levels after transfection. Real time PCR of miRs -21, -143, and -378e levels in T47D, BT549, MDA-MB-231 cells after transfection with 100nM of miRs **a.** Real time PCR of miRs -21, -143, and -378e levels in T47D cells after transfection with 200nM of anti-miRs **b.** Real time PCR of miRs -21, -143, and -378e levels in NF #6 exosomes after transfection with 130nM of miRs **c.**
